# Conventional Near-Infrared Spectroscopy and Hyperspectral Imaging: Similarities, Differences, Advantages, and Limitations

**DOI:** 10.3390/molecules30122479

**Published:** 2025-06-06

**Authors:** Daniel Cozzolino

**Affiliations:** Centre for Nutrition and Food Sciences (CNAFS), Queensland Alliance for Agriculture and Food Innovation (QAAFI), The University of Queensland, Brisbane, QLD 4072, Australia; d.cozzolino@uq.edu.au

**Keywords:** near infrared, hyperspectral imaging, big data, spectroscopy, chemometrics

## Abstract

Although, the use of sensors is increasing in a wide range of fields with great success (e.g., food, environment, pharma, etc.), their uptake is slow and lower than other innovations. While the uptake is low, some users, producers, and service industries are continuing to benefit from the incorporation of technology in their business. Among these technologies, vibrational spectroscopy has demonstrated its benefits and versatility in a wide range of applications. Both conventional near-infrared (NIR) spectroscopy and hyperspectral imaging (HSI) systems are two of the main techniques utilized in a wide range of applications in different fields. These techniques use the NIR region of the electromagnetic spectrum (750–2500 nm). Specifically, NIR-HSI systems provide spatial information and spectral data, while conventional NIR spectroscopy provides spectral information from a single point. Even though there is a clear distinction between both techniques in terms of their benefits, confusion still exists among users about their similarities and differences. This paper provides a critical discussion of the main advantages and limitations of both techniques, focusing on food science applications.

## 1. Introduction

Since the 1990s, developments in sensing technologies have been increasing, and a wide range of applications have been reported along different steps of the production, storage, and transport stages of different types of samples, including food ingredients and products [1,2,3,4,5,6]. Maximizing the potential use of different sensing technologies available in the market will provide data and information that can be incorporated into advancing food production systems alongside distribution, supply, and value chains [7,8,9,10]. These technologies will bring a shift in the way foods are evaluated and monitored at the different levels of the value and supply chains, as required to fulfil the requirements of a modern and agile food manufacturing industry. While the uptake of these technologies has been slow, users, producers and service industries are continuing to benefit from the incorporation of technology in their business [11,12]. Developments in hardware, software, communications, and sensors have allowed for improvements in existing analytical methods and techniques as well as boosting other monitoring capabilities. These developments have led to a myriad of tools being available to be utilized in the different steps of the food supply and value chain (e.g., the real-time monitoring of processes and storage conditions of food, measurement and composition) [1,2,3,4,5,6]. Furthermore, the incorporation of sensing technologies has provided agile and objective tools that enhance the information collected about either a single sample or the system itself. These tools are contributing to improving management decision systems, where sensor networks based on wireless communication, the Internet of Things (IoT), and clouds have demonstrated a high capability of sensing the safety and composition of foods from farm to fork [13,14,15].

It has been demonstrated that unlike conventional instrumentation and analytical methods, automated systems can manage a great amount of information, contributing to the generation of so-called big data [16,17,18,19,20,21,22]. However, understanding the diversity and complexity of food properties (e.g., composition, functionality, nutritive value, safety, process monitoring) as well as food systems is still required. These challenges have prompted the development of sensing technologies and expert systems [16,17,18,19,20,21,22]. Despite their potential, these emerging technologies require further research efforts to meet online inspection and quality requirements imposed by the industry and consumers [16,17,18,19,20,21,22].

Developments in sensing technologies have also been possible due to the availability of hand-held and portable equipment that can be implemented in the farm, packinghouse, storage, or even at the market to support automated processes and decision-making [23,24,25]. Recently, portability has also been extended to near-infrared (NIR) and NIR hyperspectral (HSI) systems, where a wide range of portable HSI systems have become available in the market and used in a wide range of applications (e.g., agriculture, environment, and food) [23,24,25]. Furthermore, portable and on-site sensing networks that integrate information recording, processing, analysis, and management become a reality in the so-called modern food manufacturing industry [16,17,18,19,20,21,22].

The development and implementation of the different sensing technologies available are increasing across several fields (e.g., agriculture, manufacturing industry, food, and pharmaceutical areas) with remarkable success. However, as stated in the previous sections, the overall uptake is slow. The expansion and inclusion of sensing technologies by different industries, including the food manufacturing industry, is determining a change in the classical approach, moving from the acquisition and utilization of data from individual devices in isolation into highly interconnected smart systems. These interconnected systems will reflect information from the whole process of production and supply and value chains [11,12]. Concomitantly, with the growth of data (big data) and utilization of the IoT, clouds, and networks, knowledge about the supply and value chain is increasing, allowing for better management of the food system [19,20].

With the advent of embedded devices and sensors, food systems have even started to generate massive data throughout the different steps of the system, and this vast accumulation of data has pressed the food sector into the so-called big data era [16,17,18]. Similar to many other fields, the food sector is not an exception to the so-called digital revolution. Consequently, the food manufacturing industry is dealing with an increase in data arising from the utilization of both sensing and digital technologies along the supply and value chains [19,20]. Arguably, the increase in the accessibility and utilization of sensing technologies and data is changing the current status quo in various analytical fields by strengthening existing statistical and algorithmic methods or even making traditional analytical systems redundant. Data originating from the analysis of food using sensors integrated with communication systems are typically large, heterogeneous, and dynamic [19,20].

The food manufacturing industry has been recognized as a data-driven community. Nowadays, the increase in demand for highly nutritious, sustainable, and safe foods is of high priority for the modern food manufacturing industry and the consumer [21,22]. However, this has been hindered by the complexity of the food production and supply chains, in addition to the effect of climate change (e.g., the chemical composition and nutritive value of raw materials and ingredients and physical properties), the increasingly ageing population, food safety and security, and changing patterns of consumer choice and food consumption. Due to this complexity, a better understanding as well as a rapid response to the subtle nuances influencing the production system by considering the above-mentioned factors is needed. It is in this context that the utilization of sensing technologies is enhancing the ability to harness rapid and reliable information (through big data, the use of algorithms and modelling, and the IoT) to better analyze food production systems [21,22].

Most of the existing sensing technologies used to evaluate and monitor food ingredients and products belong to the group of techniques under vibrational spectroscopy. Among these techniques, conventional NIR spectroscopy and, more recently, hyperspectral imaging (HSI) systems are the most widely utilized [23,24,25,26,27,28,29,30]. Even though there is a clear distinction between both techniques in terms of their benefits, confusion exists among users of these techniques.

This paper provides a critical discussion of the main advantages and limitations of both techniques, focusing on food science applications. Please note that this is not a review of the applications of both techniques but rather a critical and open discussion about their similarities, differences, and advantages.

## 2. Near-Infrared and Hyperspectral Imaging

Both conventional NIR spectroscopy and NIR-HSI systems utilize the NIR region of the electromagnetic spectrum (750 to 2500 nm) [31,32,33,34,35,36,37]. The utilization of NIR-HSI systems provides spatial information concomitantly with spectral data, offering what is defined as a chemical “image” of a sample, whereas conventional NIR spectroscopy collects and provides spectral information from a single point [31,32,33,34,35,36,37].

The different instruments available in the market offer different capabilities and analytical alternatives, depending on the region of the electromagnetic spectrum incorporated into the device or spectrophotometer [31,32,33,34,35,36,37]. In this way, both conventional NIR instruments and NIR-HSI systems offer the possibility to collect spectra in the visible range (VIS: 400–750 nm), including short waves in the NIR region (SWNIR: approx. 750–1200 nm) and long waves in the NIR region (approx. 1000 to 2500 nm), or combinations of these ranges depending on the instrument supplier [31,32,33,34,35,36,37]. Most of the current conventional NIR and NIR-HSI systems have been developed on indium gallium arsenide (InGaAs) detectors [31,32,33,34,35,36,37]. InGaAs detectors are capable of collecting spectra in the wavelength region approximately between 900 and 1700 nm. On the other hand, mercury cadmium telluride detectors are capable of collecting information from the full NIR wavelength range between approximately 1000 and 2500 nm [31,32,33,34,35,36,37]. The utilization of both NIR-HSI and SWIR-HSI systems is rapidly expanding in a wide range of fields, ranging from agriculture to food quality and safety applications [31,32,33,34,35,36,37].

Specifically, NIR-HSI systems have been developed to integrate both spectroscopic and imaging techniques into one system, providing the ability to simultaneously collect spectral and spatial information [31,32,33,34,35,36,37]. This unique characteristic of the NIR-HSI system has been granted to collect information from a wide range of chemical components within a sample as well as to identify or map the spatial distribution of these components within the sample. In this way, a compositional gradient or map of a given sample or product can be created [31,32,33,34,35,36,37]. On the other hand, in conventional NIR spectroscopy, one measure collects the average spectrum of the sample, while thousands of spectra can be collected with an NIR-HSI system [31,32,33,34,35,36,37]. These advantages provided by the NIR-HSI system provide possibilities to define the “whole picture” of the sample, showing that the distribution of chemical compounds can be built at the pixel level [31,32,33,34,35,36,37].

**Figure 1:**
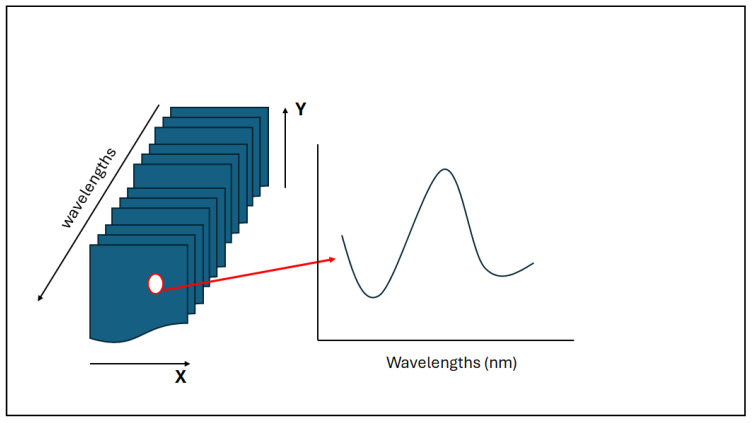
A schematic representation of the hyperspectral imaging system indicating how, from one pixel, a whole spectrum can be recreated.

As described in the previous sections, the NIR-HSI system combines spectroscopy with the recording of digital imaging [38]. In this manner, the hyperspectral image or hypercube is built on three-dimensional multivariate data. The different pixels collected in the hypercube provide the spectral responses that are associated with the chemical composition of the sample. In addition, the physical properties of the sample of interest are also collected by measuring light absorption and scattering at each of the pixels [38]. Consequently, the hyperspectral image or hypercube provides information about the measurement of the light intensity where two dimensions (X and Y) represent the spatial position, while the third dimension (wavelengths) characterizes the spectral variation in the sample analyzed (see Figure 1) [38,39,40,41,42,43,44]. The resulting image is interpreted as a stack of several two-dimensional spatial images collected at different wavelengths, aligned in rows and columns [38]. Thus, the utilization of HSI systems provides the ability to obtain high-quality spectra of surfaces that can be linked to the internal properties and chemical information of the sample [38,39,40,41,42,43,44].

**Figure 2:**
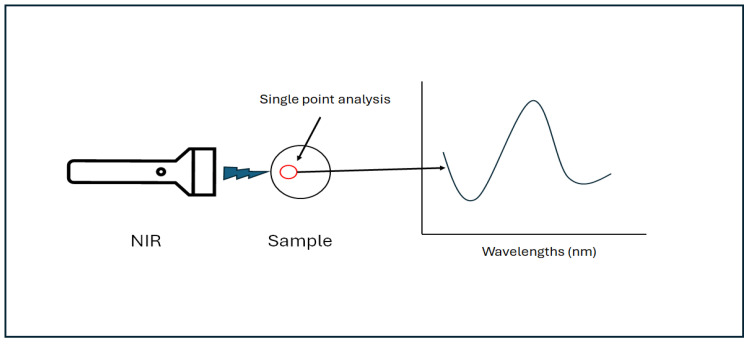
A schematic representation of conventional NIR spectroscopy indicating the collection of the spectrum from a single point.

In conventional NIR spectroscopy, large areas can also be examined without collecting multiple spectra using a large spot size or by moving the sample using a rotating sample holder (Figure 2) [39,40]. However, NIR-HSI systems are provided with more suitable options to analyze samples or materials that can be considered heterogeneous. This is possible because NIR-HSI systems can scan larger areas more quickly than single-point measurements [39,40]. Furthermore, the current commercial NIR-HSI systems available offer the possibility of collecting hyperspectral images using three basic systems, namely the (a) point (staring) scan, (b) push-broom (line) scan, or (c) plane (whiskbroom) scan [39,40]. These methods are defined by the orientation of the scanning dimension relative to the two-dimensional spatial sample axes [39,40].

## 3. Advantages and Limitations of Conventional NIR Spectroscopy and HSI Systems

The so-called conventional NIR spectroscopy, as well as NIR-HSI systems, have common advantages. These advantages include the ease of data collection, the inexpensive cost per analysis, the possibility of the rapid and simultaneous analysis of several compounds or chemical properties within the samples, and the non-destructive nature of both techniques. It is important to highlight that most of the advantages of conventional NIR spectroscopy are mirrored by those available in the NIR-HSI systems [41,42,43,44,45,46,47].

Regardless of the advantages and the analytical potential of NIR-HSI systems, their wide application has been limited due to the price of equipment [45]. Furthermore, for rapid image acquisition and data analysis, NIR-HSI systems require a high computing speed, which is considered one of the main factors limiting the utilization of this technology [45]. Both conventional NIR spectroscopy and NIR-HSI systems are indirect methods where chemometrics is required to process the data and to develop calibration models [41,42,43,44,45,46,47].

Typically, a conventional NIR spectroscopy application has been based on spectra collected using benchtop instruments where the sample (e.g., whole fruit and ground material, etc.) is positioned in front of the instrument beam or conventional NIR spectrophotometer (e.g., a rotating cup and fibre optic probes) [45]. The conventional NIR analysis offers high spectral intervals; however, the spectral information is restricted to a relatively small spatial area or single point. This may be considered a disadvantage if large and heterogeneous samples require analysis. This makes it difficult for a spectrophotometer to adequately represent within-sample variations without collecting the spectra from multiple sampling locations.

Some other limitations or disadvantages concerning these techniques include the effect of environmental variables such as temperature and moisture on the performance of the NIR-HSI cameras. Generally, these factors are more easily controllable using conventional NIR spectroscopy. Because of this, NIR-HSI cameras must be placed in controlled semi-sealed chambers when they are used in specific applications [45]. This makes the utilization of NIR-HSI camera systems more expensive when they are used in/online applications. Furthermore, NIR-HSI camera systems tend to be more sensitive to vibrations. Another limitation is associated with the speed of analysis [45]. It is well known that in conventional NIR spectroscopy, probes are particularly fast, while NIR-HSI systems still need to achieve the fast collection of spectral data with the same spectral resolution. Table 1 summarizes the advantages and limitations of both conventional NIR spectroscopy and HSI systems.

## 4. Data Analysis—Chemometrics and Machine Learning

In both conventional NIR spectroscopy and NIR-HSI systems, the development of qualitative and quantitative models requires the utilization of chemometrics and, more recently, AI tools [40,41,42,43,44]. The well-known issues that need to be considered during model development (e.g., calibration) and data processing (e.g., derivatives), such as the time taken, the availability of the software required to analyze and interpret the data, the selection of samples into calibration and validation categories, the overfitting of the models, and the need for specialists to develop a given calibration, are common for both techniques [40,41,42,43,44,45,46,47].

One of the disadvantages associated with the use of NIR-HSI systems is the collection of a series of successive overlapping bands that determine the high dimensionality of the data sets. Because of this, it is difficult to assign these bands to specific chemical groups. In addition, the existence of the so-called bad pixels can create distortions not directly associated with the information from the sample [40,41,42,43,44,45,46,47]. To identify and detect different unambiguous spectra in the same image, it is important that the samples have the same absorption characteristics [40,41,42,43,44,45,46,47].

More recently, a common strategy used in NIR-HSI systems, as well as in conventional NIR spectroscopy is to decrease the number of wavelengths collected to increase the speed of measurement during the analysis or select wavelengths using different algorithms after data collection [40,41,42,43,44,45,46,47]. This strategy could have impacted not only the quality of the signal (SNR = signal-to-noise ratio) in both conventional NIR spectroscopy and NIR-HSI systems but also the complexity of the models developed [40,41,42,43,44,45,46,47]. Critical to the development of applications using these technologies has been the trade-off between spectral resolution and SNR. For example, the utilization of NIR-HSI systems offers finer spectral resolution; however, it might sacrifice the SNR compared to conventional NIR spectroscopy. On the other hand, conventional NIR spectroscopy has lower spectral resolution but often displays a higher SNR.

Another important issue concerning the large amount of data collected is associated with the overfitting of the models. Due to the high dimensionality of the data collected and the utilization of NIR-HSI systems, the calibration models developed can have a high risk of overfitting.

To overcome the limitations and issues experienced due to the high dimensionality of the data, the integration of chemometrics and machine learning (ML) techniques, like principal component analysis (PCA) and partial least squares (PLS) regression, has provided comprehensive tools with the ability to analyze the data. The incorporation of chemometrics tools has enabled the identification of dominant trends in the data set, the classification of samples, and the ability to develop predictive models. For example, PCA has been a useful tool in reducing data dimensionality and visualizing trends, etc. In recent years, other algorithms have also been explored and reported, including support vector machines (SVMs), deep learning, artificial neural networks (ANNs), K-Nearest neighbours (KNNs), or convolutional neural networks (CNNs). Please note that it is beyond the objective of this review to provide detailed information about these algorithms where detailed information can be found elsewhere [42,43,48,49,50,51,52]. It is important to highlight that during the application or implementation of ML tools, the selection of the algorithm is not the only important step (e.g., PLS or ANN), but other variables such as the sampling method, the selection of samples and replicates, and the validation method used (e.g., cross-validation vs. independent validation) are key elements that need to be considered during the development and interpretation of the ML models [42,43,48,49,50,51,52].

## 5. What Defines the Choice Between Conventional NIR and Hyperspectral Systems?

In addition to the technical differences between conventional NIR spectroscopy and HSI systems, the target application must be considered. The scientific literature and white papers published by the vendors indicated that the NIR-HSI system can be utilized in several applications. However, several questions need to be addressed before fully embracing this technology. Is the NIR-HSI system a valid and economical option to predict or measure the chemical compound of interest in a sample (e.g., protein, dry matter)? Is the performance of the calibration model based on either conventional NIR spectroscopy or NIR-HSI systems the same? What are the improvements in terms of standard errors (calibration, cross-validation, and prediction) achieved? What do we want to achieve by using either technique?

It is not doubted that for many applications, the utilization of NIR-HSI systems does not show any clear advantage compared with the utilization of conventional NIR spectroscopy. Several applications have shown comparable performance regarding calibration statistics (standard error) for a wide range of chemical parameters, including the prediction of total soluble solids and sugars, pH, and dry matter content in fruits [31,49,52,53,54], proximate composition (e.g., protein and fat) and other quality properties in cereal grains [49,55,56], meat composition, and quality [57,58,59,60,61]. The same can be said of applications comparing the use of conventional NIR spectroscopy with NIR-HSI systems in applications targeting food authenticity, provenance, and contamination where the performance is very similar [32,40,62].

A recent study compared conventional NIR spectroscopy with NIR-HSI systems, reporting that the calibration statistics obtained using the NIR-HSI system were comparable with those obtained with conventional NIR spectroscopy [63]. This study also highlighted that when a similar wavelength range was used for all the instruments alongside the same sample sets, the NIR-HSI line-scan system worked just as well as a classical NIR spectroscopy, where the analytical time required decreased by at least half [63]. In addition, this paper showed that using open cells increased the number of samples that could be analyzed over time, allowing the development of a methodology for on-line analysis [63]. The authors showed that the main drawback of the NIR-HSI system is the loss of stability over time, which affects the performance of the HSI systems in quantitative studies; however, this can be solved using an appropriate calibration strategy. Despite this, the cost of the system is still considered one of the main limitations of the utilization of this technique for quantification. Information about the specific application of NIR-HSI systems in agriculture and food sciences can be found in the scientific literature and is beyond the objective of this review [48,49,50,51,52,53,54,55,56,57,58,59,60,61,62,63,64,65,66,67,68,69,70,71,72,73,74,75,76,77].

However, without doubt, the main advantages of the utilization of NIR-HSI systems are associated with the analysis of the distribution of chemicals or compounds in a sample, including the evaluation of patterns in the sample associated with the level of physical damage made to the sample [66], including microbiological contamination [67,71,73], or even differences in composition in mixtures or complex (heterogenous) samples [26,36,45,46].

A recent paper has objectively compared conventional NIR spectroscopy with the NIR-HSI system to analyze heterogeneous samples [45]. In this study, the authors demonstrated that heterogenous samples and the distribution of ingredients in the sample can be determined using either conventional NIR spectroscopy or NIR-HSI systems camera [45]. Interestingly, these authors concluded that the heterogeneity of the sample can be handled using conventional NIR spectroscopy (through diffuse reflectance of the spot) by collecting single-spot spectra and carefully planning the acquisition set up using a novel approach (e.g., a black/white paper template of the size of the sampling device, with decreasing black areas) [45]. Changes in the measurement set up include adjusting the focal distance, allowing a larger sample area to be covered and evaluated by the authors. Furthermore, these authors highlighted that the findings of this study would have a great impact from the process analytics technology (PAT) perspective by evaluating the ability of different types of instruments to handle heterogenous samples using easy and simple explorative analysis [29,45,72]. The same approach can be used to evaluate the economic impact of using different types of instruments in PAT applications [45,72].

In addition to the food applications described above, the pharmaceutical industry has also evaluated the utilization of NIR-HSI systems in a diverse range of applications [78,79,80,81]. Other fields have also evaluated and incorporated the utilization of NIR-HSI systems, including nanotechnology, polymers, and materials [82,83,84].

In summary, in conventional NIR spectroscopy, the balance between spot size vs. sampling representativeness can be achieved by obtaining several scans of the same sample, rotating the sample, or other combinations depending on the type of instrument utilized.

## 6. Final Considerations

The utilization and implementation of NIR spectroscopy and NIR-HSI systems as methods to evaluate and monitor the composition and safety of foods involve the combination of spectroscopy and chemometrics. The growing number of applications and the different research groups have demonstrated the benefits of both conventional NIR spectroscopy and NIR-HSI systems as tools to evaluate and monitor the composition and safety of foods. Yet, the routine and commercial implementation of these technologies is still not widely embraced by the food manufacturing industry.

The heterogeneity of the sample is still one of the most common arguments that is utilized to justify the application of the NIR-HSI system over conventional NIR spectroscopy. However, its evaluation or comparison with other sample presentation alternatives offered by conventional NIR spectroscopy is not evaluated or reported (e.g., the use of NIR probes and rotating cups, etc.). Although this justification is generally true, there are situations where conventional NIR spectroscopy is a valid technical option compared to NIR-HSI systems.

Most of the scientific literature in the field has described feasibility studies of both conventional NIR spectroscopy and NIR-HSI systems, where small data sets or calibration models are reported without proper independent validation. Furthermore, other variables, including a lack of in-depth understanding of the reference method (e.g., chemical analysis) used to develop the models (e.g., standard errors of the reference or laboratory method), the poor or null design of experiments, the wide range of issues associated with the sampling and selection of samples to be used during calibration and validation, the poor or no interpretation of significance variables (e.g., the analysis and interpretation of loadings or coefficients of regression), and the poor understanding of the existence of inter-correlations among variables (e.g., wavelengths and variables/parameters measured, or those between different parameters), are still hindering the proper comparison and utilization of both techniques. In addition, the lack of objective comparisons (e.g., a proper statistical analysis) of the coefficient of determination in either calibration or validation (R^2^) analysis, standard errors (e.g., calibration, cross-validation, and prediction) or the classification rates (e.g., the percentage of correct classification) obtained mislead current and potential users of this technology.

The use of chemometrics, machine learning (ML) or AI aids to resolve and gather the most relevant information about the entire food system with important bits that might remain uncovered. No matter the extent and incorporation of new chemometric tools, methods, or techniques (e.g., new algorithms, new software, and mathematical pre-processing), during spectra collection, issues such as best sample presentation, processing, or interactions of the sample collection and analysis can occur and are not considered. The characteristics of the instrument (conventional NIR spectroscopy vs. NIR-HSI systems) and properties of the sample, together with the operating conditions and carelessness expressed during the implementation of NIR spectroscopy, are often ignored by current and potential users of the technology. Although the versatility of both conventional NIR spectroscopy and NIR-HSI systems offers the ability to analyze different samples in different conditions (e.g., the presence of dust, elevated temperatures, humidity conditions, and vibration during the analysis), care should be taken. Overall, the net benefits of the application of conventional NIR spectroscopy are dissimilar to those obtained using HSI depending on the measurement of the same compositional parameters at different locations of the food production and value chain. Furthermore, both conventional NIR spectroscopy and HSI systems can be used in heterogenous samples; however, they have significant differences in terms of time and cost, whereas conventional NIR spectroscopy (single point) offers a faster and less expensive solution, making it ideal for rapid and less detailed analysis.

In addition, the implementation of both conventional NIR spectroscopy and NIR-HSI systems to evaluate and monitor the chemical composition, safety, and any other issues associated with biosecurity in food ingredients and products requires the integration of skilled human resources with knowledge in a wide range of fields (e.g., spectroscopy, analytical chemistry, data analytics, chemometrics, biology, and physics). Consequently, the development and implementation of these techniques requires different strategies. One such approach followed by different research groups is fostering collaboration with the food industry as a key participant in the R&D process. In this partnership, it is important to demonstrate the cost benefits (e.g., reduction in cost, energy, and time) of applying these technologies in an industrial setting, their contribution towards the development of agile and objective management decision systems and tools, and their ability to monitor changes in the process and consistency of food production, or even the ability to provide information or tools that contribute to reducing food waste along the value chain. It will be important to highlight that most of this task and its benefits are in addition to or go beyond the reporting of calibration statistics.

Finally, the amalgamation of these resources defines the utilization of these techniques as multidisciplinary. Beyond the simple selection of a technique, these tools should be considered as an important component of a system that provides data and information that contribute to agile and smart decision management tools.

## Figures and Tables

**Table 1:** A summary of the advantages and limitations of hyperspectral imaging systems compared with conventional NIR spectroscopy.

	HSI System	Conventional NIR Spectroscopy
**Cost**		
Cost of Instrumentation	High	High to low
Cost Per Analysis	Inexpensive	Inexpensive
**Data Collection**		
Data Collection	Easy to collect	Easy to collect
Data Analysis and Interpretation	Requires chemometrics and data pre-processing	Requires chemometrics and data pre-processing
**Sample collection**	Non-destructive analysis	Non-destructive analysis
**Speed of analysis**	Rapid and simultaneous analysis of the sample	Rapid and simultaneous analysis of the sample
**Measurement**	Spectra and spatial information	Single-point spectra
**Type of samples**	Heterogeneous	Homogenous ^¥^
**Analysis**	Map or image showing the distribution of compounds in the sample analyzed	No map or distribution and no detailed information provided about the distribution of the compounds in the sample

HSI: hyperspectral imaging; NIR: near-infrared. ¥ Please note that sample presentation can provide analytical alternatives to analyze heterogenous samples.

## Data Availability

No new data were created or analyzed in this study.
